# Association between acetabular dysplasia and sagittal spino-pelvic alignment in a population-based cohort in Japan

**DOI:** 10.1038/s41598-022-16865-1

**Published:** 2022-07-25

**Authors:** Teiji Harada, Hiroshi Hashizume, Takaya Taniguchi, Toshiko Iidaka, Yoshiki Asai, Hiroyuki Oka, Shigeyuki Muraki, Toru Akune, Hiroshi Kawaguchi, Kozo Nakamura, Munehito Yoshida, Sakae Tanaka, Noriko Yoshimura, Hiroshi Yamada

**Affiliations:** 1 Department of Orthopaedic Surgery, Wakayama Medical University, 811-1 Kimiidera, Wakayama City, Wakayama Japan; 2 School of Health and Nursing Science, Wakayama Medical University, 590 Mikazura, Wakayama City, Wakayama Japan; 3 Department of Preventive Medicine for Locomotive Organ Disorders, 22nd Century Medical & Research Center, Faculty of Medicine, The University of Tokyo, 7-3-1 Hongo, Bunkyo-ku, Tokyo, Japan; 4 Department of Medical Research and Management for Musculoskeletal Pain, 22nd Century Medical & Research Center, Faculty of Medicine, The University of Tokyo, 7-3-1 Hongo, Bunkyo-ku, Tokyo, Japan; 5 National Rehabilitation Center for Persons with Disabilities, 4-1 Namiki, Tokorozawa City, Saitama Japan; 6 Department of Orthopaedics and Spine, Tokyo Neurological Center, 4-1-17 Toranomon, Minato-ku, Tokyo, Japan; 7 Department of Orthopaedic Surgery, Towa Hospital, 4-7-10 Towa, Adachi-ku, Tokyo, Japan; 8 Department of Orthopaedic Surgery, Sumiya Orthopedic Hospital, 337 Yoshida, Wakayama City, Wakayama Japan; 9 Department of Orthopaedic Surgery, Faculty of Medicine, The University of Tokyo, 7-3-1 Hongo, Bunkyo-ku, Tokyo, Japan

**Keywords:** Epidemiology, Osteoarthritis, Skeleton

## Abstract

The relationship between acetabular dysplasia and spino-pelvic alignment remains unclear. The aim of this study was to clarify the association between acetabular dysplasia and spino-pelvic alignment, based on a large-scale population-based cohort in Japan. From the third survey of the Research on Osteoarthritis/Osteoporosis Against Disability (ROAD) study, 1,481 participants (491 men and 990 women; mean age, 65.3 years) were analyzed. Center-edge (CE) angle and spino-pelvic parameters (lumbar lordosis, LL; sacral slope, SS; pelvic tilt, PT; pelvic incidence, PI) were measured radiographically. Acetabular dysplasia was defined as a CE angle < 20°, and associations between acetabular dysplasia and spino-pelvic parameters were assessed. The group with acetabular dysplasia had significantly higher age, higher percentage of female, higher SS and higher PI than the group without acetabular dysplasia in a univariate analysis. On the other hand, acetabular dysplasia was not significantly associated with spino-pelvic parameters in a multiple logistic regression analysis that include age, sex, SS and PI as explanatory variables; however, PI demonstrated a positive odds ratio (odds ratio, 1.02; 95% CI 1.00–1.04). In conclusion, acetabular dysplasia was not significantly associated with spino-pelvic parameters, but higher PI may be an associated factor for acetabular dysplasia.

## Introduction

Offierski and MacNab^1^ first described the significant relationship between the hip joints and spine as the hip-spine syndrome in 1983. It is important to clarify the influence of spino-pelvic alignment for understanding the pathophysiology of hip diseases. Acetabular dysplasia is a major factor associated with osteoarthritis (OA) of the hip^2–4^. In Japan, majority of the hip OA cases are secondary to acetabular dysplasia^5^. Poor coverage over the femoral head is caused by the shallow and oblique shape of the acetabulum. This feature is commonly observed in acetabular dysplasia, thus inducing excessive stress on the articular cartilage and subsequently resulting in hip joint instability and OA^6,7^. Patients with acetabular dysplasia show anterior inclination of the pelvis to increase their acetabular coverage^6,8,9^. However, the role of spino-pelvic alignment in acetabular dysplasia remains unclear.

The purpose of this study was to clarify the association between acetabular dysplasia and spino-pelvic alignment, based on a large-scale population-based cohort in Japan.

## Materials and methods

### Participants

The Research on Osteoarthritis/Osteoporosis Against Disability (ROAD) study is a large-scale population-based cohort study of bone and joint diseases established in three communities in Japan: an urban region in Itabashi, Tokyo, with a population of 529,400/32 km^2^; a mountainous region in Hidakagawa, Wakayama with a population of 11,300/330 km^2^; and a coastal region in Taiji, Wakayama with a population of 3500/6 km^2^. The detailed profile of the ROAD study had been previously reported^10–13^. This study included participants from the third survey of the ROAD study involving the mountain and coastal regions.

The third survey of the ROAD study was conducted between October 2012 and December 2013. In addition to the previous participants, inhabitants of the mountainous and coastal areas in the Wakayama prefecture who were willing to participate in the study were also included. Among the total 1575 individuals (513 men and 1062 women) who participated in the survey, 59 individuals who did not undergo hip or whole-spine radiography and 13 individuals who had indistinct radiographs or other disqualifiers were excluded. Additionally, 22 individuals with a history of hip surgery due to OA or proximal femoral fracture were excluded owing to the changes in hip morphology. The remaining 1481 participants (491 men and 990 women; mean age, 65.3 years) were included in this study. The characteristics of the participants are listed in Table 1.

**Table 1:** Demographics of the participants.

	Total	Men	Women
Number of participants	1481	491	990
Age (years)	65.3 ± 13.0	66.2 ± 13.8	64.9 ± 12.5
Height (cm)	156 ± 9.1	165 ± 7.2	152.0 ± 6.6
Weight (kg)	56.4 ± 11.3	64.6 ± 11.4	52.4 ± 8.7
BMI (kg/m^2^)	23.0 ± 3.5	23.7 ± 3.5	22.7 ± 3.5

Values are presented as mean ± standard deviation (95% confidence intervals).

*BMI* body mass index.

The participants documented their family medical history, personal medical history, and hip surgery history through an interviewer-administered questionnaire. Physical parameters including height and weight were recorded, from which body mass index (BMI) was calculated. Furthermore, all participants were assessed by well-experienced orthopedists for the presence of hip pain, which was defined as the presence of any hip pain for most part of the past month in addition to current pain.

### Radiographic evaluation

**Figure 1:**
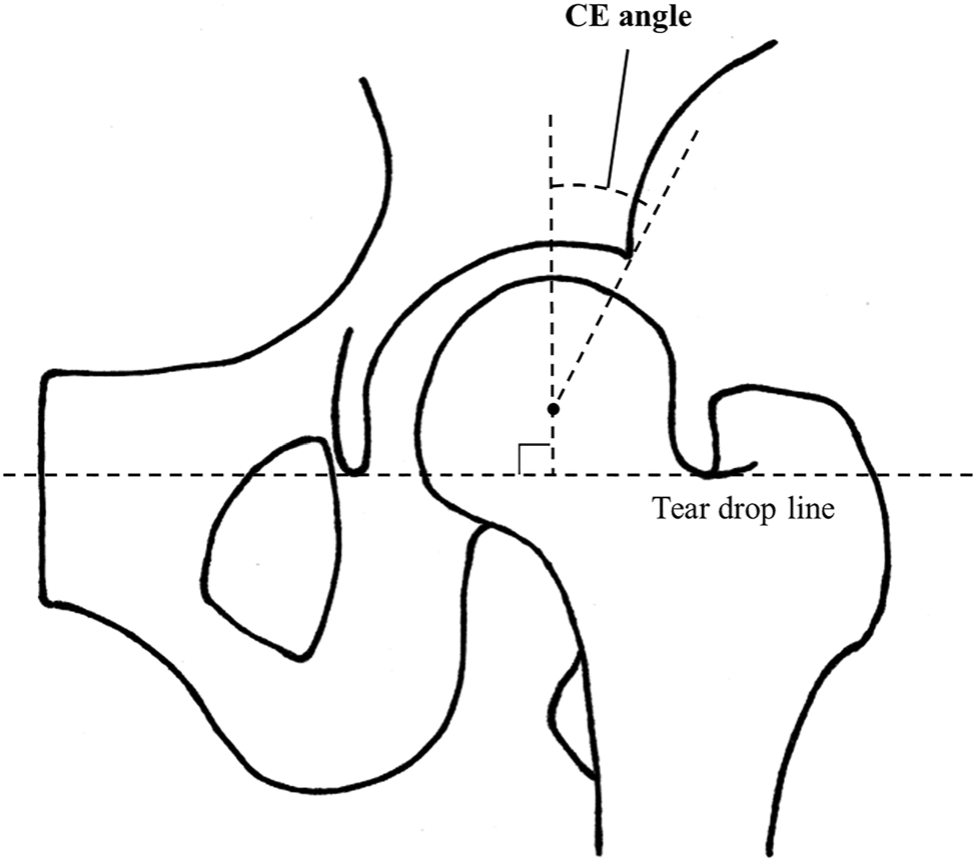
Measurement of the center-edge (CE) angle. The angle formed by a vertical line drawn from the center of the femoral head perpendicular to the teardrop line and a line drawn from the center of the femoral head to the lateral edge of the acetabulum is measured.

All participants underwent radiographic examinations. Anteroposterior radiography of both hips was performed under weight-bearing conditions. Hip radiographs were assessed by a single well-experienced orthopedist (T.H.) who was blinded of the participants’ clinical status, and the center edge (CE) angle was measured. The CE angle was defined as the angle formed by a vertical line drawn from the center of the femoral head perpendicular to the teardrop line and a line drawn from the center of the femoral head to the lateral edge of the acetabulum (Fig. 1)^14^. In this study, acetabular dysplasia corresponded to a CE angle < 20°, and the participants with acetabular dysplasia in either or both hips were classified as the presence of acetabular dysplasia.

**Figure 2:**
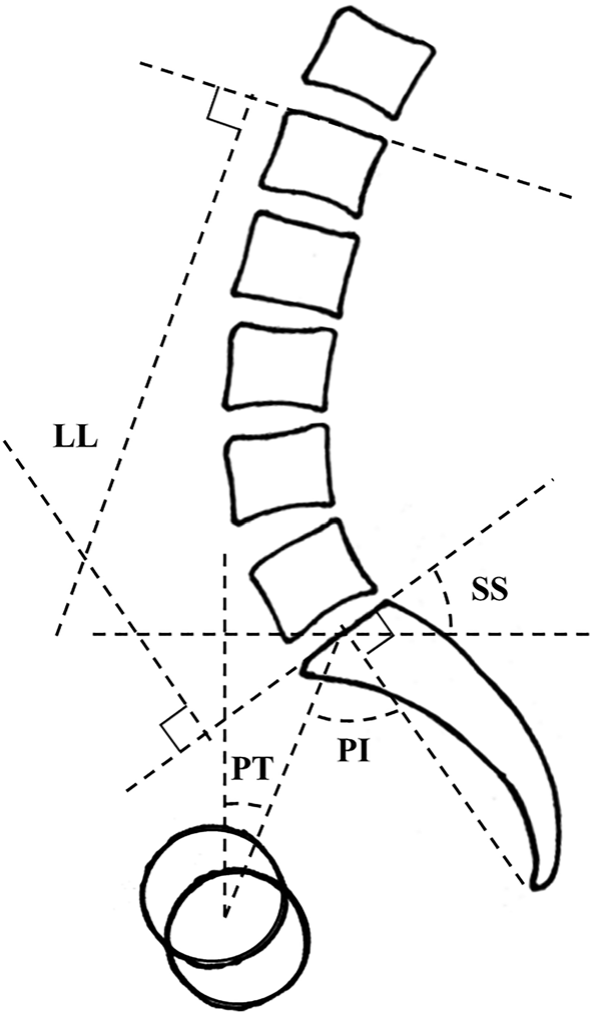
Measurement of sagittal spino-pelvic parameters. *LL* lumbar lordosis, *SS* sacral slope, *PT* pelvic tilt, *PI* pelvic incidence.

Standing lateral radiography of the whole spine and pelvis was also performed with the edge of the film as a reference for vertical alignment. The following spino-pelvic parameters of sagittal spino-pelvic alignment were measured on the radiographs: lumbar lordosis (LL; Cobb angle from the upper endplate of L1 to the upper endplate of S1), sacral slope (SS; the angle between the tangent line to the upper endplate of S1 and the horizontal plane), pelvic tilt (PT; the angle between the line connecting the midpoint of the sacral plate to the axis of the femoral heads and the vertical axis), pelvic incidence (PI; the angle between the line perpendicular to the sacral plate at its midpoint and the line connecting this point to the axes of the femoral heads) (Fig. 2)^15,16^.

To evaluate the intraobserver reliability of the CE angle and spino-pelvic parameters, each 50 randomly selected hip and whole spine radiographs were assessed by the same observer more than 1 month after the first measurement, while another 50 radiographs were analyzed by two experienced orthopedists (CE angle, T.H. and T.I.; spino-pelvic parameters, T.H. and Y.A.) to evaluate the interobserver reliability. The intraobserver and interobserver reliability based on intraclass coefficients of correlation was adequate for assessment (CE angle, 0.96 and 0.85; LL, 0.96 and 0.94; SS, 0.91 and 0.93; PT, 0.99 and 0.97; PI, 0.93 and 0.91; respectively).

### Statistical analysis

Statistical analyses were performed using the JMP software (version 15; SAS Institute Inc., Cary, NC, USA). Quantitative values are expressed as the mean ± standard deviation (SD) and 95% confidence intervals (CIs). The chi-square test was used to compare the presence of acetabular dysplasia and the presence of hip pain related to acetabular dysplasia between men and women. Differences in age, height, weight, BMI, and sagittal spino-pelvic parameters (LL, SS, PT, and PI) between groups with and without acetabular dysplasia were evaluated using the non-paired t-test. In addition, these differences were evaluated between groups with and without hip pain in the acetabular dysplasia group. Multiple logistic regression analysis was conducted to examine the association of spino-pelvic parameters with acetabular dysplasia. The explanatory variables were those that reached statistical significance in the univariate analysis. Odds ratios were provided with 95% CIs.

### Ethics declarations

All participants provided written informed consent for their participation and for the publication of the study in print and electronic form.

This study, establishment of the cohort and study design, was approved by the Research Ethics Review Committee of the University of Tokyo (No. 1326). The procedures followed were in accordance with the ethical standards of the responsible committee on human experimentation (institutional and national) and with the Helsinki Declaration of 1975, as revised in 2000.

## Results

### Prevalence of acetabular dysplasia

The prevalence of acetabular dysplasia was 42/491 (8.6%) in men and 144/990 (14.5%) in women. It was significantly higher in women than in men (P = 0.0011).

### Comparison of the demographics and spino-pelvic parameters between the groups with and without acetabular dysplasia

The demographics and spino-pelvic parameters classified by participants with and without acetabular dysplasia are shown in Table 2. The group with acetabular dysplasia comprised a significantly higher proportion of women and participants of younger age than the group without acetabular dysplasia. Regarding the spino-pelvic parameters, SS and PI were significantly higher in the group with acetabular dysplasia (P = 0.0291, P = 0.0092, respectively), while LL and PT revealed no significant difference between these two groups.

**Table 2:** Comparison of the demographics and spino-pelvic parameters between the groups with and without acetabular dysplasia.

Number of participants	Acetabular dysplasia (+)	Acetabular dysplasia (−)	P value
Demographics	186	1295	
Sex (men vs women)	42 vs 144	449 vs 846	0.0011*
Age (years)	62.3 ± 15.0 (60.2–64.5)	65.8 ± 12.6 (65.1–66.4)	0.0054^†^
Height (cm)	155 ± 8.2 (153.8–156.1)	157 ± 9.2 (156.0–157.0)	0.0507
Weight (kg)	54.8 ± 10.0 (53.4–56.3)	56.4 ± 11.4 (56.0–57.3)	0.0878
BMI (kg/m^2^)	22.8 ± 3.4 (22.3–23.3)	23.0 ± 3.5 (22.8–23.2)	0.5350
**Spino-pelvic parameters**			
LL (°)	46.0 ± 14.4 (44.0–48.1)	45.4 ± 13.6 (44.6–46.1)	0.4852
SS (°)	33.2 ± 9.0 (31.9–34.5)	31.6 ± 9.1 (31.0–32.1)	0.0291^†^
PT (°)	18.7 ± 9.2 (17.4–20.0)	18.1 ± 9.2 (17.6–18.6)	0.3626
PI (°)	51.9 ± 10.9 (50.3–53.4)	49.7 ± 10.6 (49.1–50.2)	0.0092^†^

Results are presented as mean ± standard deviation (95% confidence intervals).

*BMI* body mass index, *LL* lumbar lordosis, *SS* sacral slope, *PT* pelvic tilt, *PI* pelvic incidence.

*Significant group difference by chi-square test.

^†^Significant group difference by non-paired t-test.

### Association of acetabular dysplasia with spino-pelvic parameters

Multiple logistic regression analysis with the presence/absence of acetabular dysplasia as the objective variable and age, sex, SS, and PI as explanatory variables, which were significantly different in univariate analysis was conducted to investigate the association between spino-pelvic parameters and acetabular dysplasia. The presence of acetabular dysplasia significantly associated with younger age and female sex. There was no significant association between acetabular dysplasia and spin-pelvic parameters; however, PI demonstrated a positive odds ratio (odds ratio, 1.02; 95% CI 1.00–1.04) (Table 3).

**Table 3:** Association between the presence of acetabular dysplasia and spino-pelvic parameters.

	Reference	Odds ratio	95% CI	P value
Age	+ 1 year	0.98	0.97–0.99	0.0014*
Sex	Women (vs. men)	1.66	1.14–2.41	0.0076*
SS	+ 1°	1.00	0.98–1.02	0.8743
PI	+ 1°	1.02	1.00–1.04	0.0589

Odds ratios are calculated by multiple logistic regression analysis on 1481 participants.

*95% CI* 95% confidence interval, *SS* sacral slope, *PI* pelvic incidence.

*Statistically significant association with acetabular dysplasia.

### Association of hip pain related to acetabular dysplasia with spino-pelvic parameters

In the group with acetabular dysplasia, the presence of hip pain was detected in 10/186 (5.4%) participants. The group with hip pain related to acetabular dysplasia comprised significantly higher height than the group without hip pain (Table 4). No significant difference was observed between the presence of hip pain related to acetabula dysplasia and spino-pelvic parameters (Table 4).

**Table 4:** Comparison of the demographics and spino-pelvic parameters between the groups with and without hip pain in participants with acetabular dysplasia.

Number of participants/hips	Hip pain (+)	Hip pain (−)	P value
Demographics	10	176	
Sex (men vs women)	0 vs 10	42 vs 134	0.0791
Age (years)	59.0 ± 11.6 (50.7–67.3)	62.5 ± 15.2 (60.3–64.8)	0.3961
Height (cm)	151.0 ± 3.8 (148.3–153.7)	155 ± 8.4 (153.9–156.4)	0.0470*
Weight (kg)	56.7 ± 13.6 (47.0–66.4)	54.7 ± 9.8 (53.3–56.2)	0.8657
BMI (kg/m^2^)	24.8 ± 5.4 (20.9–28.7)	22.7 ± 3.2 (22.2–23.2)	0.2893
**Spino-pelvic parameters**			
LL (°)	41.7 ± 10.4 (34.3–49.1)	46.3 ± 14.5 (44.1–48.4)	0.2653
SS (°)	30.6 ± 9.1 (24.1–37.1)	33.3 ± 9.0 (32.0–34.6)	0.3895
PT (°)	18.9 ± 6.6 (14.2–23.7)	18.7 ± 9.3 (17.3–20.1)	0.7696
PI (°)	49.6 ± 11.7 (41.2–57.9)	52.0 ± 10.9 (50.4–53.6)	0.4064

Results are presented as mean ± standard deviation (95% confidence intervals).

*BMI* body mass index, *LL* lumbar lordosis, *SS* sacral slope, *PT* pelvic tilt, *PI* pelvic incidence.

*Significant group difference by non-paired t-test.

## Discussion

To the best of our knowledge, this is the first study to clarify the association between acetabular dysplasia and spino-pelvic alignment, based on a large-scale population-based cohort. The prevalence of acetabular dysplasia was significantly associated with younger age and female sex, consistent with previous report^13^.

Regarding the effect of spino-pelvic parameters on acetabular dysplasia, Matsuyama et al.^8^ reported that the compensatory anterior inclination of the pelvis and lumbar hyperlordosis were observed in patients with bilateral congenital hip dislocation. Fukushima et al.^6^ compared sagittal spino-pelvic parameters in female patients with and without acetabular dysplasia. They reported significantly higher SS and LL in patients with acetabular dysplasia. These reports suggested that the anterior inclination of the pelvis in patients with acetabular dysplasia, which compensates for the poor acetabular coverage, might be concomitant with lumbar hyperlordosis. In this study, SS and PI were significantly higher in the group with acetabular dysplasia than the group without acetabular dysplasia in the univariate analysis. On the other hand, there was no significant association between the presence of acetabular dysplasia and spino-pelvic parameters in the multiple logistic regression analysis. However, PI demonstrated a positive odds ratio (odds ratio, 1.02; 95% CI 1.00–1.04), although it was not statistically significant (p = 0.0589), indicating that higher PI may be an associated factor for acetabular dysplasia. Higher PI may be related with morphological features of dysplastic hips, in which the femoral heads tend to be located superiorly than normal hips.

In the group with acetabular dysplasia, there was no significant difference in demographic data, except for height, between the groups with and without hip pain. Although the chi-square test that compared the presence of hip pain with acetabular dysplasia between men and women showed no significant difference, all participants with hip pain related to acetabular dysplasia were women. This may be the reason for the significant difference in height. Furthermore, no significant difference was observed between the hip pain related to acetabular dysplasia and sagittal spino-pelvic parameters. Offierski and MacNab classified the pathology of hip-spine syndrome into four categories^1^. In the first category, secondary hip-spine syndrome was described as the presence of a major lesion in the hip joints or spine, which affected each other. For example, lumbar degenerative kyphosis causes posterior inclination of the pelvis, resulting in poor anterior coverage of the acetabulum, which may lead to primary OA of the hip in older adults^17–19^. Alternatively, Okuda et al.^9^ reported that patients with hip OA secondary to acetabular dysplasia maintained lumbar lordosis and anterior inclination of the pelvis, regardless of aging. In patients with acetabular dysplasia, spino-pelvic alignment changes due to aging might have little effect on the development of OA of the hip.

This study had several limitations. First, because the ROAD study was instituted to establish the epidemiological indices of degenerative diseases of locomotive organs, a high proportion of the participants were older adults. Thus, the effects of degenerative changes on the spine due to aging could not be ruled out and accurate results might not have been obtained pertaining to the association between acetabular dysplasia and functional spino-pelvic parameters such as LL, SS, and PT. Second, acetabular coverage was assessed only by measuring the CE angle on anteroposterior radiography of both hips under weight-bearing conditions. Moreover, pelvic inclination alters the measures of CE angle^20^. Therefore, radiographic measurements may have underestimated or overestimated the acetabular coverage. Magnetic resonance imaging or computed tomography for three-dimensional imaging can be used in future for the proper assessment of acetabular coverage. Third, the small number of participants with hip pain related to acetabular dysplasia in this study was not sufficient to clarify the association of the hip pain with spino-pelvic alignment. Finally, a selection bias may exist. Since the participants with a history of hip surgery were excluded from this study, patients with severe OA or other lesions of the hip might not have been included. Further investigation of a large population of younger people is needed to assess the effect of spino-pelvic alignment on the development of OA of the hip in patients with acetabular dysplasia.

In conclusion, the large-scale population-based cohort study revealed the association between acetabular dysplasia and sagittal spino-pelvic parameters in a general Japanese population. Acetabular dysplasia was not significantly associated with spino-pelvic parameters, but high PI may be an associated factor for acetabular dysplasia. Higher PI may be related with morphological features of dysplastic hips, in which the femoral heads tend to be located superiorly than normal hips.

## Data Availability

All data generated or analyzed during this study are available from the corresponding author upon reasonable request.
